# Effectiveness of oral motor interventions on feeding and orofacial motor outcomes in preterm infants: a systematic review and meta-analysis of randomized controlled trials

**DOI:** 10.1186/s12887-026-06905-4

**Published:** 2026-05-06

**Authors:** Rina Febriani Puspitasari, Putri Kusuma Wardani Mahendra, Anrizandy Narwidina

**Affiliations:** 1 Specialist Study Program of Pediatric Dentistry, Faculty of Dentistry, Universitas Gadjah Mada, Yogyakarta, Indonesia; 2 Department of Pediatric Dentistry, Faculty of Dentistry, Universitas Gadjah Mada, Yogyakarta, Indonesia

**Keywords:** Oral Motor Interventions, Feeding Readiness, Feeding Function, PIOMI, Developmental Delays

## Abstract

**Background:**

Efficient oral feeding is a key functional milestone in neonatal and early childhood care, reflecting neuromuscular maturation, physiological stability, and readiness for hospital discharge. Infants and young children, particularly those born preterm, frequently experience feeding difficulties due to immature oral motor coordination. Structured oral motor interventions have been proposed to enhance feeding-related outcomes; however, their effectiveness remains variably reported. This systematic review and meta-analysis aimed to evaluate the effectiveness of oral motor interventions in improving feeding and oral motor outcomes in infants and young children compared with routine care.

**Methods:**

A systematic review and meta-analysis were conducted following the Preferred Reporting Items for Systematic Reviews and Meta-Analyses 2020 guidelines. Randomized controlled trials published between 2014 and 2024 were identified through PubMed, Cochrane Library, and Google Scholar. Eligible studies involved infants and young children with feeding difficulties or underdeveloped oral motor function and evaluated structured oral motor interventions compared with routine care or standard feeding support. Study selection was performed using Rayyan. Risk of bias was assessed using the Cochrane Risk of Bias tool version 2.0. Where appropriate, random-effects meta-analyses were performed, and effect estimates were presented as mean differences or standardized mean differences with 95% confidence intervals. The certainty of evidence was evaluated using the Grading of Recommendations Assessment, Development and Evaluation approach.

**Results:**

Ten randomized controlled trials were included. All included studies involved preterm infants receiving neonatal clinical care. Oral motor interventions were associated with improvements in feeding readiness, sucking–swallowing coordination, feeding efficiency, and earlier achievement of full oral feeding compared with routine care. Interventions incorporating Premature Infant Oral Motor Intervention demonstrated the most consistent benefits, particularly in reducing the time required to achieve independent oral feeding. The certainty of evidence ranged from low to moderate, with methodological heterogeneity and limited blinding representing the main sources of bias.

**Conclusions:**

Oral motor interventions were associated with clinically relevant improvements in feeding-related outcomes among preterm infants compared with routine care. Overall, the findings support oral motor interventions as a clinically relevant adjunct to routine care for preterm infants with feeding difficulties associated with underdeveloped oral motor function. However, the certainty of evidence ranged from low to moderate, highlighting the need for further well-designed randomized controlled trials to clarify long-term developmental implications and inform evidence-based clinical practice.

**Trial registration:**

PROSPERO CRD420251270705.

## Background

Orofacial muscles play a central role in maintaining craniofacial stability and long-term functional outcomes. This relationship is well described by the triangular force concept, which emphasizes biomechanical equilibrium through the balanced distribution of muscular forces acting on the dentition, alveolar bone, and surrounding soft tissues [1, 2]. Adequate orofacial muscle function supports structural stability by maintaining balanced forces on the dentition and limiting the influence of destabilizing oral habits, such as tongue thrusting and mouth breathing. In addition, harmonious interactions among the teeth, jaws, and soft tissues contribute to functional occlusion, reduce the risk of temporomandibular joint (TMJ) disorders, and preserve facial symmetry, lip posture, and overall facial harmony as key determinants of long-term aesthetic and functional outcomes [1–3]. Preterm infants frequently experience feeding difficulties due to immature coordination of sucking, swallowing, and breathing. This physiological immaturity may lead to delayed transition from tube feeding to oral feeding and prolonged hospitalization [1, 2]. Oral motor interventions have therefore been proposed as supportive strategies to facilitate feeding readiness and neuromuscular maturation in preterm infants [3, 4].

The development of orofacial muscle strength and feeding function in infants and young children follows a predictable, age-dependent sequence. During the first six months of life, feeding relies primarily on reflexive suckling and swallowing. As neuromuscular control matures, coordinated chewing and swallowing typically emerge between 12 and 24 months. By approximately three to six years of age, children achieve near adult-like orofacial motor control, enabling efficient feeding, clear speech articulation, and the ability to manage a wide range of food textures. This progressive maturation underscores the foundational role of early orofacial muscle development in establishing normal feeding patterns, communication, and orofacial functional competence throughout early childhood [4, 5].

Despite this well-established developmental framework, gaps remain in understanding how targeted therapeutic approaches may address delays in infants and young children with underdeveloped orofacial muscles. In clinical practice, routine care generally emphasizes overall feeding support but often lacks structured, goal-directed orofacial stimulation specifically designed to enhance muscle strength, coordination, and sensorimotor integration [6]. This limitation raises important questions regarding whether oral motor interventions (OMIs) can more effectively promote orofacial muscle maturation and feeding-related milestones compared with routine care alone.

Accordingly, this systematic review aims to evaluate the effectiveness of oral motor interventions in improving orofacial muscle development and feeding function in infants and young children. By synthesizing evidence from randomized controlled trials, this review seeks to determine whether structured oral motor stimulation provides measurable benefits beyond those achieved through routine care without targeted oral motor therapy.

## Methods

### Study design and study protocol

This study was conducted as a systematic review with meta-analysis, following the Preferred Reporting Items for Systematic Reviews and Meta-Analyses (PRISMA) 2020 guidelines. The review protocol was prospectively registered in the International Prospective Register of Systematic Reviews (PROSPERO) under registration number CRD420251270705.

The aim of this systematic review was to evaluate the effectiveness of oral motor interventions (OMIs) applied in pediatric dentistry and related clinical settings on orofacial muscle development and feeding function in infants and young children.

### Eligibility criteria

Eligibility criteria were established a priori using the PICO framework. Studies were eligible if they involved infants and young children (≤ 18 years) presenting with feeding difficulties or impaired orofacial muscle function attributable to oral motor dysfunction, including both preterm and full-term infant populations. Although the eligibility criteria permitted inclusion of broader pediatric populations, all eligible randomized controlled trials identified in the final dataset involved preterm infants receiving neonatal care. Studies involving adults, children with fully developed orofacial motor function, or feeding difficulties unrelated to oral motor or neuromuscular mechanisms were excluded. Eligible studies evaluated structured oral motor interventions (OMIs) aimed at improving orofacial muscle strength, coordination, or functional feeding performance. Interventions included oral motor therapy, oral motor exercises, stimulation-based oral motor protocols, and standardized interventions such as Premature Infant Oral Motor Intervention (PIOMI) and Beckman-based oral motor techniques. Across the included studies, oral motor interventions were primarily delivered in neonatal intensive care units or neonatal inpatient wards. The interventions were typically administered by trained healthcare professionals including neonatal nurses, speech language therapists, or occupational therapists under multidisciplinary neonatal care supervision. Interventions not specifically targeting orofacial muscle function or feeding-related outcomes (e.g., general physical therapy or non-oral motor sensory stimulation) were excluded. Comparator conditions included routine nursing care, standard feeding or pediatric care without structured oral motor stimulation, sham interventions, or no intervention. Studies without a clearly defined comparator or control group were excluded. Only randomized controlled trials (RCTs) were included to ensure a high level of methodological rigor. Non-randomized studies, quasi-experimental designs, observational studies, case reports, editorials, narrative reviews, conference abstracts, and systematic reviews were excluded. Eligible studies were required to report pre- and post-intervention outcomes related to at least one of the following domains: Feeding function, including oral feeding readiness, sucking, and swallowing coordination, feeding efficiency, time to full oral feeding, or related clinical feeding milestones; and/or orofacial muscle development or function, including oral motor control, strength, coordination of lips, tongue, cheeks or jaw, assessed using validated instruments or standardized clinical measures.

For the purpose of this review, underdeveloped oral motor function refers to clinically documented feeding immaturity commonly observed in preterm infants, including poor coordination of sucking, swallowing, and breathing, weak or inconsistent non-nutritive sucking patterns, delayed transition from tube feeding to oral feeding, or clinically identified feeding difficulties requiring structured oral motor intervention.

### Search strategy and data resources

A systematic literature search was conducted using PubMed, Cochrane Library, and Google Scholar. For Google Scholar, the first 200 results sorted by relevance were screened to ensure feasibility and reproducibility of the search process. The search was restricted to studies published in English between January 2014 and December 2024 to capture contemporary evidence relevant to current pediatric dentistry and feeding intervention practices.

Search terms were developed to identify studies related to oral motor interventions and pediatric feeding outcomes and included combinations of the following keywords and their related terms: “oral motor intervention,” “oral motor therapy,” “oral motor stimulation,” “Beckman therapy,” “PIOMI,” “orofacial muscles,” “feeding function,” “feeding readiness,” “infants,” and “children.” Reference lists of all included studies were manually screened to identify additional relevant articles.

### Selection process for studies

All identified records were imported into Rayyan (Qatar Computing Research Institute), a web-based systematic review screening software, to facilitate duplicate removal and blinded screening. Duplicate records were automatically detected and removed prior to the screening process.

Titles and abstracts were independently screened by two reviewers using Rayyan according to the predefined eligibility criteria. Full-text articles of potentially eligible studies were subsequently retrieved and assessed independently by the same reviewers. Any disagreements during the screening process were resolved through discussion and consensus. The overall study selection process was documented using a PRISMA flow diagram.

### Data extraction

Data extraction was performed independently by two reviewers using a standardized data extraction form. Extracted information included study characteristics, participant demographics, intervention details, comparator conditions, outcome measures, and main results. Discrepancies in data extraction were resolved through discussion. Study authors were not contacted for additional information when data were incomplete or unclear.

### Risk of bias and quality assessment

The methodological quality of included randomized controlled trials was assessed using the Cochrane Risk of Bias tool version 2.0 (RoB 2.0). The assessment addressed five domains: (1) bias arising from the randomization process, (2) bias due to deviations from intended interventions, (3) bias due to missing outcome data, (4) bias in measurement of outcomes, and (5) bias in selection of the reported results.

Each domain was rated as low risk, some concerns, or high risk, and an overall risk of bias judgment was assigned for each study. Assessments were conducted independently by two reviewers, with disagreements resolved by consensus.

The certainty of evidence for key outcomes was evaluated using the GRADE approach.

### Data integration and analysis

A narrative synthesis was conducted to summarize findings across included studies. Where studies were sufficiently homogeneous in terms of participants, interventions, and outcome measures, a quantitative synthesis (meta-analysis) was performed using Review Manager (RevMan) software.

Continuous outcomes were pooled using mean differences (MD) or standardized mean differences (SMD) with corresponding 95% confidence intervals, depending on outcome measurement scales. A random-effects model was applied to account for clinical and methodological heterogeneity. Statistical heterogeneity was assessed using the I² statistic, and results were presented using forest plots. Formal assessment of publication bias using funnel plots or Egger’s test was not performed because fewer than 10 studies contributed to each pooled outcome.

## Results

### Study selection

The systematic literature search conducted across PubMed, Cochrane Library, and Google Scholar for studies published between 2014 and 2024 yielded a total of 62 records. After removal of duplicate records, all remaining studies were imported into Rayyan for screening. Several studies involving term infants or mixed infant populations were identified during the initial screening process but were excluded during full text review due to non-randomized study design, absence of structured oral motor intervention, or lack of relevant feeding related outcomes.

**Fig. 1:**
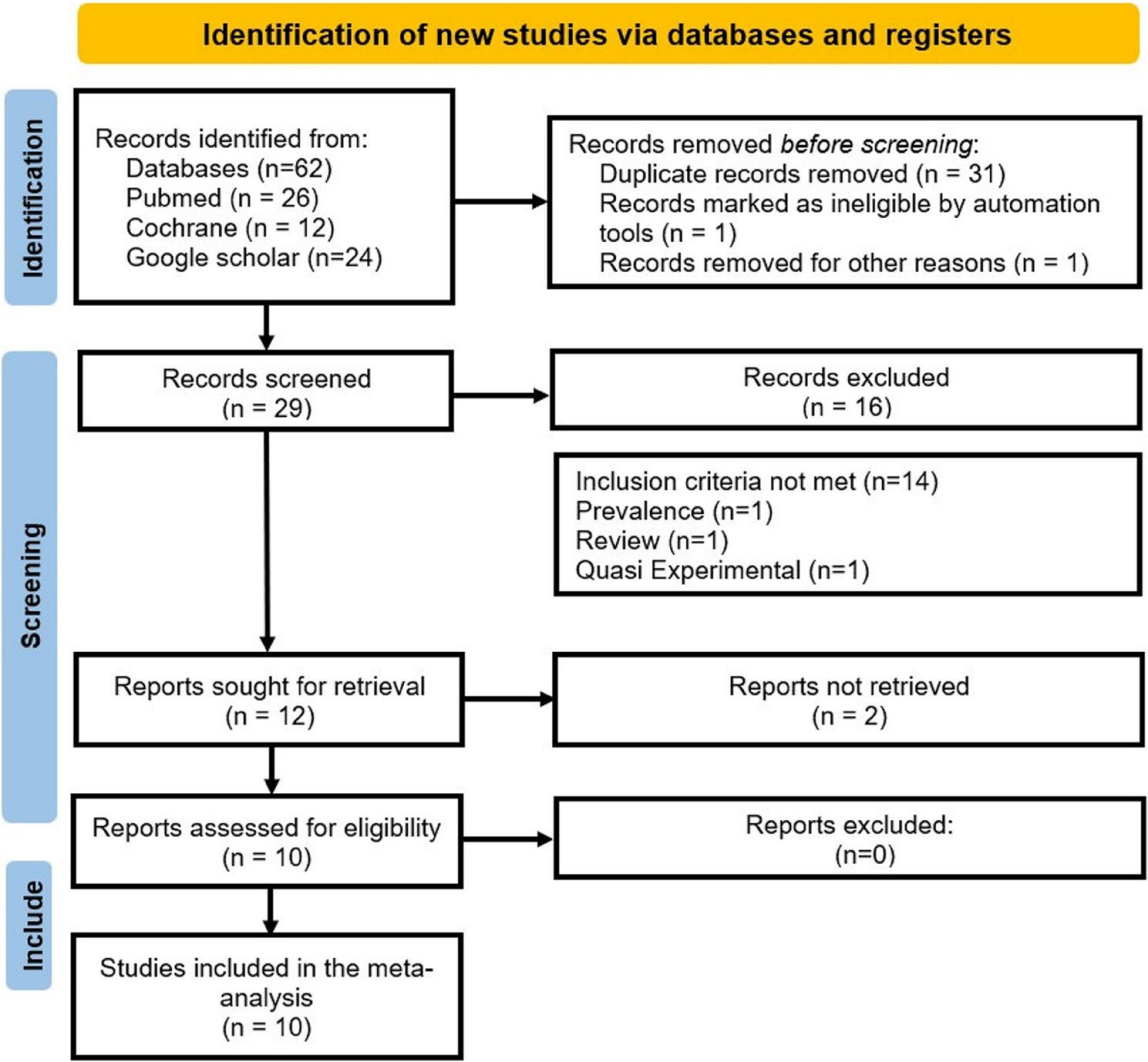
PRISMA flow diagram of study selection. Flow diagram illustrating the study selection process according to PRISMA 2020 guidelines. The diagram shows the identification of records from electronic databases, removal of duplicates and ineligible records, screening of titles and abstracts, assessment of full-text articles for eligibility, and final inclusion of randomized controlled trials in the meta-analysis

Title and abstract screening resulted in the exclusion of 33 records that did not meet the predefined eligibility criteria, primarily due to irrelevant outcomes, inappropriate study design, or non-pediatric populations. A total of 29 full-text articles were assessed for eligibility. Following full-text evaluation, 19 studies were excluded for reasons including non-randomized study design, absence of a control group, interventions not classified as structured oral motor interventions, or outcomes unrelated to feeding function or orofacial muscle development. Ultimately, 10 randomized controlled trials fulfilled the inclusion criteria and were included in the qualitative synthesis, with all eligible for quantitative synthesis. The detailed study selection process is illustrated in the PRISMA flow diagram (Fig. 1**).** Two reports were classified as not retrieved because their full texts could not be accessed despite attempts through institutional databases and manual search strategies; therefore eligibility could not be determined.

### Study characteristics

The characteristics of the included studies are summarized in Table 1. All 10 included studies employed a randomized controlled trial design and were conducted in neonatal or pediatric clinical settings.

**Table 1:** Characteristics of the 10 included randomized controlled trials

Study (Year)	Country	Study design	Population / Sample size	Intervention	Comparator	Duration	Main feeding readiness / oral motor outcomes	Main feeding performance outcomes
Arora et al. (2018) [7]	India	Randomized clinical trial	Preterm infants; PIOMI (*n* = 16), Control (*n* = 14)	PIOMI	Routine care	7 days	Improved NOMAS scores and oral motor organization	Earlier attainment of independent oral feeding
Ghomi et al. (2019) [8]	Iran	Randomized clinical trial	Preterm infants; PIOMI (*n* = 28), Control (*n* = 28)	PIOMI	Routine care	10 days	Improved oral feeding readiness and coordination	Faster transition to full oral feeding
Lessen Knoll et al. (2019) [9]	Iran	Randomized clinical trial	Preterm infants; PIOMI (*n* = 15), Control (*n* = 15)	PIOMI	Routine care	7 days	Improved oral motor organization and feeding readiness behaviors	Earlier initiation and shorter transition to full oral feeding
Mahmoodi et al. (2019) [10]	Iran	Randomized clinical trial	Preterm infants (sample size not specified by sex)	PIOMI	Routine care	7 days	Enhanced feeding readiness and coordination	Faster progression to oral feeding milestones
Güler et al. (2022) [11]	Turkey	Randomized clinical trial	Preterm infants; PIOMI (*n* = 30), Sham (*n* = 30)	PIOMI	Sham intervention	14 days	Improved sucking power, duration, and rhythm	Shorter time to full oral feeding and reduced hospital stay
Li et al. (2022) [4]	China	Randomized clinical trial	Preterm infants; OMI + NNS (*n* = 30), NNS (*n* = 30)	OMI combined with NNS	NNS alone	14 days	Higher POFRAS-CV scores indicating improved feeding readiness	Greater milk intake and faster transition to oral feeding
Ghazi et al. (2023) [12]	Iran	Randomized clinical trial	Preterm infants; GA ≈ 37 weeks	PIOMI (15-min oral stimulation)	Routine care	7 days	Improved feeding readiness scores	Faster transition to oral feeding and improved efficiency
Shokri et al. (2023) [13]	Iran	Randomized clinical trial	Preterm infants	PIOMI combined with music therapy	PIOMI alone	10 days	Greater improvement in feeding readiness scores	Shorter transition time to oral feeding
Singh et al. (2023) [3]	India	Randomized clinical trial	Preterm infants; PIOMI (*n* = 42), Control (*n* = 42)	Structured PIOMI	Unstructured oral motor stimulation	7 days	Significantly higher POFRAS scores	Earlier progression to independent oral feeding
Huang et al. (2024) [14]	Taiwan	Randomized clinical trial	Preterm infants	PIOMI	Routine care	7 days	Higher NOMAS-based feeding readiness scores	More efficient feeding performance and shorter transition time

Abbreviations: *PIOMI* Premature Infant Oral Motor Intervention, *OMI* Oral Motor Intervention, *NNS* Non-nutritive sucking, *NOMAS* Neonatal Oral Motor Assessment Scale, *POFRAS-CV* Preterm Oral Feeding Readiness Assessment Scale–Chinese Version, *GA* gestational age

The study populations consisted primarily of infants and young children, including both preterm and full-term participants presenting with feeding difficulties associated with underdeveloped oral motor function. Sample sizes varied across studies.

All studies evaluated structured oral motor interventions (OMIs), with the Premature Infant Oral Motor Intervention (PIOMI) being the most frequently investigated protocol. Other interventions included PIOMI derived from Beckman-based oral motor techniques and structured oral stimulation programs. Intervention durations ranged from 7 to 14 days, with daily intervention sessions administered under controlled clinical conditions.

Comparator groups received routine or standard care without structured oral motor stimulation. Outcome measures focused on two primary domains: (1) feeding-related outcomes, including feeding readiness, sucking, and swallowing coordination, time to achieve full oral feeding, feeding efficiency, and milk intake; and (2) orofacial or oral motor function outcomes, assessed using validated tools such as the Neonatal Oral Motor Assessment Scale (NOMAS), Premature Oral Feeding Readiness Assessment Scale (POFRAS), or structured clinical assessments. Across the included studies, feeding related outcomes were generally assessed immediately after completion of the intervention period or at the time of transition to full oral feeding, which frequently coincided with readiness for discharge from neonatal inpatient care. Variability in outcome timing across studies has been acknowledged.

### Risk of bias within studies

**Fig. 2:**
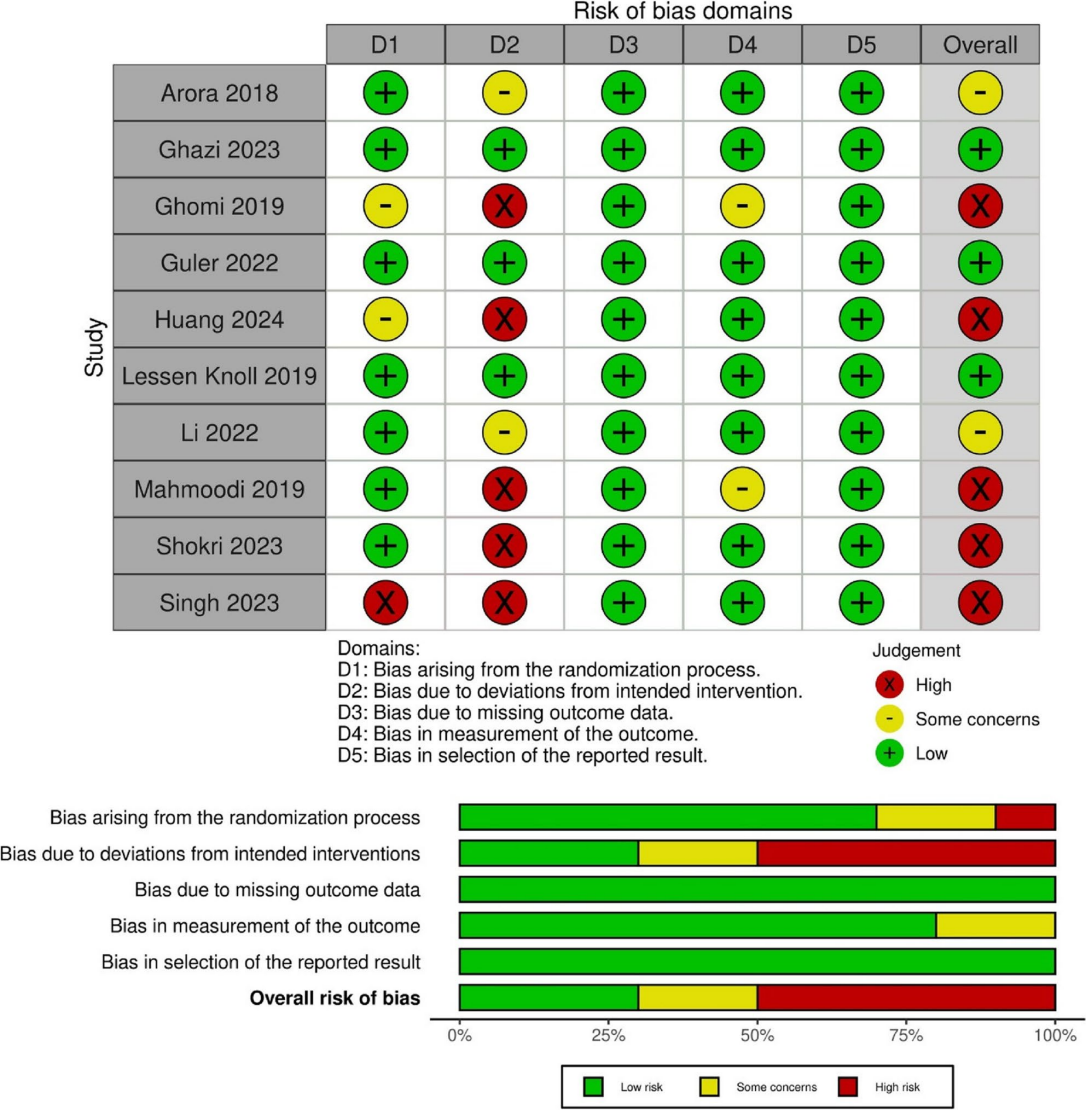
Risk of bias assessment of included studies. Risk of bias assessment of the included randomized controlled trials using the Cochrane Risk of Bias 2.0 tool. Individual studies are evaluated across five domains: bias arising from the randomization process (D1), bias due to deviations from intended interventions (D2), bias due to missing outcome data (D3), bias in measurement of the outcome (D4), and bias in selection of the reported result (D5). Overall risk of bias judgments for each study and summary proportions across domains are presented

The risk of bias assessment for individual studies is presented in Fig. 2, based on the Cochrane Risk of Bias tool version 2.0 (RoB 2.0). Among the included studies, three trials were judged to have a low risk of bias, two trials raised some concerns, and five trials were assessed as having a high risk of bias. Bias arising from the randomization process was generally low, although some studies provided limited details regarding allocation concealment. The most frequent source of bias was related to deviations from intended interventions, primarily due to insufficient reporting of adherence to intervention protocols or lack of blinding of caregivers and outcome assessors. In contrast, missing outcome data and selection of the reported result were judged to be at low risk across all included studies, indicating adequate completeness and transparency of outcome reporting. Overall, while methodological quality varied across studies, outcome measurement and reporting were generally consistent.

### Synthesis of results

Quantitative synthesis was conducted for outcomes reported by at least one randomized controlled trial with comparable outcome measures. The results are presented as forest plots using a random-effects model, and stratified according to intervention type and risk of bias where applicable.

#### Oral feeding outcome (PIOMI vs. standard care)

**Fig. 3:**
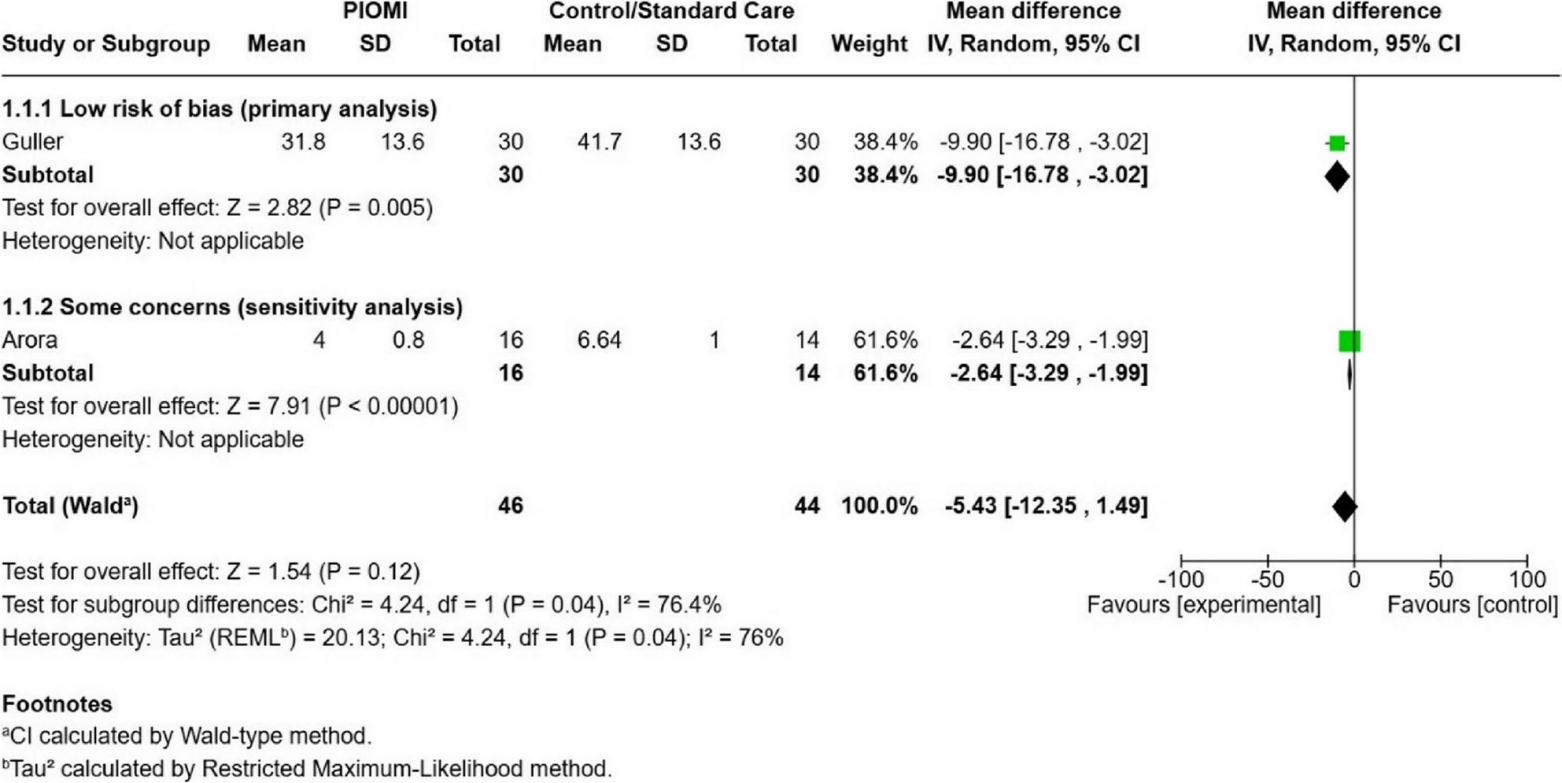
Forest plot of oral feeding outcomes (PIOMI vs. standard care). Forest plot comparing oral feeding outcomes between Premature Infant Oral Motor Intervention (PIOMI) and standard care. Results are presented as mean differences with 95% confidence intervals using a random-effects model. Subgroup analyses are shown according to overall risk of bias categories. The direction of effect indicates favor towards the intervention or control group as displayed in the plot

The effect of Premature Infant Oral Motor Intervention (PIOMI) on oral feeding outcomes compared with standard care is presented in Fig. 3. In the primary analysis including studies with low risk of bias, PIOMI demonstrated a statistically significant improvement in oral feeding outcomes compared with standard care (mean difference [MD] = − 9.90; 95% CI: −16.78 to − 3.02; *p* = 0.005). This finding indicates a clinically relevant reduction in time to achieving effective oral feeding [11].

A sensitivity analysis including studies with some concerns regarding risk of bias also showed a significant benefit of PIOMI (MD = − 2.64; 95% CI: −3.29 to − 1.99; *p* < 0.00001) [7]. However, when all eligible studies were pooled, the overall effect was no longer statistically significant (MD = − 5.43; 95% CI: −12.35 to 1.49; *p* = 0.12), with substantial heterogeneity observed between subgroups (I² = 76%). These findings suggest that while PIOMI may improve oral feeding outcomes, the magnitude and consistency of the effect appear to be influenced by study quality and methodological differences [7, 11].

#### Feeding efficiency outcome (oral motor intervention plus NNS vs. NNS alone)

**Fig. 4:**
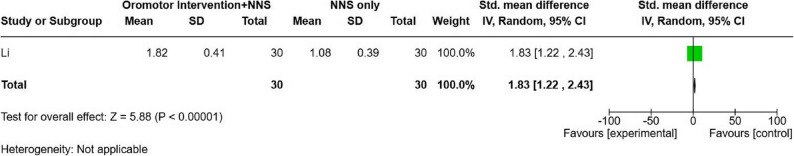
Forest plot of feeding efficiency outcomes (OMI plus NNS vs. NNS alone). Forest plot comparing feeding efficiency outcomes between oral motor intervention combined with non-nutritive sucking (OMI + NNS) and non-nutritive sucking alone. Standardized mean differences with 95% confidence intervals were calculated using a random-effects model. The plot displays individual study estimates and the pooled effect size

The effect of combined oral motor intervention and non-nutritive sucking (NNS) compared with NNS alone on feeding efficiency is illustrated in Fig. 4.

The pooled analysis from one randomized controlled trial demonstrated a large and statistically significant improvement in feeding efficiency favoring the combined intervention (standardized mean difference [SMD] = 1.83; 95% CI: 1.22 to 2.43; *p* < 0.00001). No statistical heterogeneity was observed [4].

This result indicates that the addition of structured oral motor stimulation to NNS may provide substantial benefits in enhancing feeding performance compared with NNS alone.

#### Feeding readiness outcome (oral motor intervention vs. standard care)

**Fig. 5:**
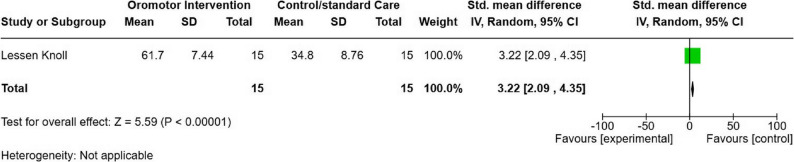
Forest plot of feeding readiness outcomes (OMI vs. standard care). Forest plot comparing feeding readiness outcomes between oral motor intervention and standard care. Standardized mean differences with 95% confidence intervals are presented using a random-effects model. The direction of the effect favors the intervention or control group as indicated in the plot

The effect of oral motor intervention on feeding readiness compared with standard care is presented in Fig. 5. A single randomized controlled trial demonstrated a significant improvement in feeding readiness scores favoring the intervention group (SMD = 3.22; 95% CI: 2.09 to 4.35; *p* < 0.00001). No heterogeneity was observed due to the inclusion of a single study [9].

This finding suggests that structured oral motor intervention may markedly enhance feeding readiness in infants, although further studies are required to confirm the robustness and generalizability of this effect.

### Risk of bias across studies

The certainty of evidence across outcomes was assessed using the Grading of Recommendations, Assessment, Development and Evaluation (GRADE) approach. This assessment considered study limitations, inconsistency, indirectness, imprecision, and potential publication bias. The summary of findings and certainty ratings for the main outcomes are presented in Table 2.

**Table 2:** Summary of findings for oral motor interventions compared with standard care

Outcome	No. of participants (studies)	Effect	Certainty of evidence (GRADE)	What happens
Time to full oral feeding (days)	90 (2 RCTs)	Mean difference − 5.4 days (95% CI − 12.4 to 1.5)	⨁⨁⨁◯ Moderate	Oral motor intervention may reduce the time required to achieve full oral feeding compared with standard care; however, the confidence interval includes no effect, indicating uncertainty in the estimate.
Weight gain (g/day)	60 (1 RCT)	Not pooled	⨁⨁◯◯ Low	Oral motor intervention combined with non-nutritive sucking may increase daily weight gain in preterm infants compared with non-nutritive sucking alone, based on evidence from a single randomized controlled trial.
Feeding efficiency (% of prescribed intake)	30 (1 RCT)	Not pooled	⨁⨁◯◯ Low	Oral motor intervention may improve feeding efficiency in preterm infants, with higher percentages of prescribed oral intake consumed and faster improvement during the first days of oral feeding.

*CI* confidence interval, *GRADE* Grading of Recommendations Assessment, Development and Evaluation, *RCT* randomized controlled trial

Overall, the certainty of evidence for oral feeding outcomes comparing PIOMI with standard care was rated as moderate. Although statistically significant benefits were observed in analyses restricted to studies with low risk of bias, the overall pooled effect showed substantial heterogeneity. This inconsistency, largely attributable to differences in methodological quality and outcome measurement across studies, resulted in downgrading the certainty of evidence by one level.

For feeding efficiency outcomes comparing oral motor intervention combined with non-nutritive sucking (NNS) versus NNS alone, the certainty of evidence was rated as moderate to high. The observed effect size was large and consistent; however, the certainty was downgraded due to imprecision related to the inclusion of a single randomized controlled trial.

Similarly, the certainty of evidence for feeding readiness outcomes comparing oral motor intervention with standard care was rated as moderate. While the intervention demonstrated a large and statistically significant effect, the evidence base was limited to a single study, warranting cautious interpretation.

Across all outcomes, the most common methodological limitation was related to blinding of participants and personnel, which is inherently challenging in non-pharmacological interventions. Nevertheless, outcome measurement was generally objective and consistently reported, reducing the likelihood of detection bias. No clear evidence of publication bias was identified, although the small number of included studies limited formal assessment.

Considering the totality of evidence, the GRADE assessment indicates that oral motor interventions provide clinically relevant improvements in feeding-related surrogate neonatal outcomes, supported by moderate certainty of evidence. These findings support the potential role of structured oral motor interventions in promoting functional feeding maturity, while highlighting the need for further high-quality, adequately powered trials to strengthen confidence in long-term developmental outcomes and comparative effectiveness across intervention protocols.

## Discussion

### Summary of results

This systematic review demonstrates that oral motor interventions (OMIs) are consistently associated with improvements in feeding readiness, oral motor coordination, and functional feeding outcomes in infants and young children when compared with routine care. Across the included randomized controlled trials, infants receiving structured oral motor stimulation achieved earlier initiation of oral feeding, shorter transition times to full oral feeding, and improved sucking efficiency.

These findings are consistent with contemporary models of orofacial functional development, which emphasize the coordinated activity of the lips, tongue, jaw, and associated musculature as a foundation for effective oral function and long-term craniofacial stability [1, 2]. Recent clinical and developmental studies further support the concept that early optimization of oral motor function contributes to improved feeding performance and functional adaptation during critical periods of growth [15, 16]. Interventions such as Premature Infant Oral Motor Intervention (PIOMI) appear to facilitate neuromuscular organization during early development and are associated with clinically relevant oral improvements in feeding performance and oral motor control.

### Factors affecting the main outcome

The beneficial effects of oral motor interventions can be explained through neurodevelopmental, sensorimotor, and biomechanical mechanisms underlying early oral function. Feeding requires precise coordination of sucking, swallowing, and respiration, regulated by central pattern generators within the brainstem and refined through afferent sensory input from the oral and perioral regions [17, 18]. The coordination of sucking, swallowing, and breathing typically matures between 32 and 34 weeks of gestational age [1, 2]. Preterm infants born before this developmental milestone frequently exhibit feeding immaturity, making them particularly vulnerable to delayed oral feeding progression [3]. Oral motor interventions may therefore support neuromuscular feeding development during this critical period of neonatal neurodevelopment [4]. More recent neurodevelopmental research highlights the importance of experience-dependent neural plasticity in shaping early motor patterns, including feeding-related behaviors [19, 20].

Structured oral motor stimulation enhances sensory–motor integration by providing repetitive tactile and proprioceptive input, thereby supporting neuromuscular maturation and motor learning [15, 21, 22]. Contemporary studies emphasize that targeted and developmentally appropriate stimulation, rather than passive oral input, is more effective in promoting coordinated sucking and swallowing, particularly in infants with immature neuromuscular control [16, 23].

Intervention characteristics also influence outcomes. Protocols such as the Premature Infant Oral Motor Intervention (PIOMI), which apply a standardized, brief, and sequential pattern of oral stimulation specifically adapted to the anatomical and neurodevelopmental characteristics of preterm infants, are consistently associated with earlier feeding readiness, faster transition to full oral feeding, and improved feeding-related outcomes compared with routine or unstructured oral motor stimulation [3, 7, 8, 11, 13, 14]. Early initiation and consistent application of oral motor therapy appear to enhance neuromuscular adaptation, supporting earlier attainment of independent oral feeding and functional oral competence [15, 24].

In the context of neonatal and early pediatric research, feeding-related milestones including feeding readiness, feeding efficiency, and time to full oral feeding are widely recognized as clinically meaningful surrogate neonatal outcomes [16, 23]. These measures reflect the integrated maturation of orofacial muscle strength, neuromuscular coordination, respiratory stability, and sensorimotor control that underpin safe and effective oral feeding. Although surrogate outcomes do not directly capture long-term neurodevelopmental trajectories, they provide pragmatic and clinically relevant indicators of functional maturity, early adaptation, and readiness for discharge [15]. Accordingly, improvements in feeding-related outcomes observed following oral motor interventions may be interpreted as early markers of broader functional competence during critical periods of neonatal and infant development.

### Other considerations

In addition to immediate feeding outcomes, oral motor interventions may have broader implications for orofacial functional development and adaptation. Contemporary literature suggests that early oral motor experiences influence subsequent abilities related to mastication, speech articulation, and oral sensory processing, which depend on coordinated orofacial muscle activity and neuromuscular integration [1, 2].

Improved feeding competence has also been associated with shorter hospital stays, reduced dependence on enteral feeding, and improved caregiver–infant interaction, outcomes that are increasingly recognized as indicators of quality of care in neonatal and pediatric settings [15, 25]. Importantly, oral motor interventions remain non-invasive, low-cost, and clinically feasible, supporting their integration into routine multidisciplinary care when delivered using standardized protocols and appropriate training [23].

### Implications for pediatric and neonatal care

From a broader health system perspective, the findings of this review have important implications for neonatal and pediatric healthcare worldwide. Delays in achieving full oral feeding remain a major contributor to prolonged neonatal intensive care unit (NICU) length of stay (LOS), increased healthcare costs, and heightened caregiver burden, particularly in preterm populations [15, 23].

Earlier attainment of independent oral feeding has been consistently associated with shorter hospitalization, reduced reliance on enteral feeding, and improved caregiver–infant interaction, all of which are recognized quality indicators in neonatal care [16]. By facilitating more rapid feeding readiness and feeding competence, structured oral motor interventions may contribute to reduced NICU burden and more efficient resource utilization, especially in low- and middle-income settings where prolonged hospitalization may pose substantial economic and logistical challenges. The non-invasive, low-cost, and easily implementable nature of these interventions further supports their potential scalability and integration into routine neonatal care pathways.

### Limitations and strengths

Several limitations should be considered when interpreting the findings of this review. Because all included randomized controlled trials involved preterm infants, the findings of this meta-analysis may not be generalizable to term infants or older pediatric populations. Additionally, although all included studies employed randomized controlled designs, the overall certainty of evidence was predominantly moderate according to the GRADE assessment. This was primarily due to inconsistency and imprecision across outcomes. For oral feeding outcomes, substantial heterogeneity was observed, reflecting variability in intervention protocols, outcome definitions, and study quality. Such heterogeneity limited confidence in the pooled effect estimates and necessitated cautious interpretation of the magnitude of benefit.

Second, for feeding efficiency and feeding readiness outcomes, the evidence base was restricted to single randomized controlled trials, resulting in downgrading of certainty due to imprecision. While large effect sizes were observed, the limited number of contributing studies reduces the generalizability of these findings and highlights the need for replication in larger, well-designed trials.

Another important limitation relates to methodological constraints inherent to non-pharmacological interventions, particularly the difficulty of blinding participants and caregivers. This limitation was consistently identified in the risk of bias assessment and contributed to downgrading of the certainty of evidence. However, outcome measurement was generally objective and standardized, reducing the likelihood that lack of blinding substantially influenced outcome assessment.

Despite these limitations, this review has several notable strengths. A key strength is the inclusion of only randomized controlled trials, enhancing internal validity and reducing the influence of confounding factors. In addition, the review followed a prospectively registered protocol and adhered to PRISMA 2020 guidelines, ensuring methodological transparency and minimizing selective reporting.

Furthermore, the consistency of direction of effect across studies, particularly for feeding-related outcomes, supports the biological plausibility of the observed benefits and aligns with established principles of orofacial and neuromuscular development. Importantly, the GRADE assessment indicates that, despite moderate certainty, the observed effects are likely to be clinically meaningful, supporting the role of oral motor interventions as a valuable adjunct to routine care.

Overall, while further high-quality, adequately powered trials with standardized outcome measures are required to strengthen the certainty of evidence and clarify long-term effects, the current findings provide a robust and methodologically sound basis for the observed benefits of oral motor interventions in early childhood.

## Conclusion

Oral motor interventions were associated with clinically relevant improvements in feeding-related outcomes among infants and young children compared with routine care. Across the included randomized controlled trials, these interventions contributed to enhanced feeding readiness, improved oral feeding performance, and earlier attainment of effective oral feeding, particularly in preterm populations.

From a pediatric and neonatal care perspective, feeding-related outcomes represent important functional indicators of neuromuscular maturation and physiological readiness. Improvements in these outcomes may support more efficient feeding progression and facilitate earlier transition to independent oral feeding within clinical care settings.

Overall, the findings support oral motor interventions as a clinically relevant adjunct to routine care for infants and young children with feeding difficulties associated with underdeveloped oral motor function. However, the certainty of evidence ranged from low to moderate, reflecting methodological heterogeneity and limitations in study design. Further well-designed randomized controlled trials with standardized outcome measures and longer follow-up are needed to clarify long-term developmental implications and to inform evidence-based clinical practice.

## Data Availability

All data generated or analysed during this study are included in this published article and its supplementary information files.
